# Food allergen analysis: Considerations for establishing a reference measurement system to implement EU legislation

**DOI:** 10.1016/j.foodchem.2023.136391

**Published:** 2023-10-30

**Authors:** Elena Cubero-Leon, Hendrik Emons, Gavin O'Connor, Jørgen Nørgaard, Piotr Robouch

**Affiliations:** a European Commission, Joint Research Centre (JRC), Geel, Belgium; b Physikalisch-Technische Bundesanstalt, Bundesallee 100, 38116 Braunschweig, Germany

**Keywords:** Food allergen analysis, Comparability of results, Metrological traceability, Calibration hierarchy, Reference measurement system

## Abstract

Inconsistent quantification results obtained from various analytical methods for food allergen testing hamper an accurate quantitative risk assessment and its regulatory implementation. In order to overcome such problems, a concept aiming at ensuring the comparability of quantitative food allergen measurement results is presented here. It is based on an approach called reference measurement system for food allergens, which uses a commonly agreed reference, namely the ‘mass fraction of total protein of the allergenic ingredient in food’. The necessary system components are outlined, consisting of a primary reference measurement method, a certified reference material and a reference laboratory. This metrology-based concept can be applied to quantify various food allergens determined with different analytical procedures. The example of 'milk in cookies' is used to demonstrate the approach.

## Highlights

• Inconsistent food allergen measurement results hamper accurate quantitative risk assessments.
• Comparability of food allergen data can be achieved via reference measurement systems.
• Such an approach has been developed for quantifying total milk protein in cookies.
• Further research is needed to implement such systems for other food allergens.

## 1 Introduction

Food allergies cause significant public health concern affecting approximately 5 to 7 % of children and 1 to 2 % of adults worldwide (Ballmer-Weber et al., 2015). Currently, the only remedy for affected individuals is to avoid certain food containing ingredients to which they are allergic. Therefore, the appropriate labelling of food allergens is essential for protecting people with food allergies.

The General Food Law (Regulation (EC) No 178, 2002) of the European Union requires food business operators to implement appropriate risk assessment and risk management procedures to ensure that food placed on the market is safe. In the context of food allergens, in Europe it is mandatory to label the presence of fourteen food products or substances potentially causing allergies or intolerances when used as ingredients (Regulation (EU) No 1169, 2011). In some instances, food business operators may be unable to avoid and/or to mitigate the unintended presence of these allergens. In these cases, they can use voluntary ‘precautionary allergen labelling’ (PAL) as a communication and risk management tool. Regulation (EU) No 1169/2011 (2011) provides the legal basis for using PAL. It states that “*voluntary information shall not be ambiguous or confusing for the consumer, and shall be based on the relevant scientific data”*. However, there is still no specific guidance on the application of PAL, hence resulting in its diverse and inconsistent use. Consequently, consumers with food allergies lose trust in PAL, making it less effective in protecting them from food allergens (Soon & Manning, 2017).

Recently, progress has been made to standardise quantitative risk assessment processes and the use of PAL (Remington et al., 2022, WHO/FAO, 2021a, WHO/FAO, 2021b). However, these advances depend on the availability of ‘comparable measurement results’ for the allergen content of food. Comparability requires data availability for the same ‘quantification parameter’ for the investigated allergen. In 2017, several European stakeholders including health professionals, food allergy consumer organizations, food industry risk assessors and the food allergen testing community agreed to report the analytical results as “*mass of total protein of the allergenic food ingredient per mass of food*”, expressed in mg kg^−1^ (O'Connor & Ulberth, 2017). This “*quantity intended to be measured”*, referred to as the ‘measurand’ in the International Vocabulary of Metrology (JCGM 200, 2012), can then be directly used to assess food safety.

Agreeing on such a common measurand for risk assessment and risk management of food allergens is crucial for obtaining comparable data. However, transforming analytical data derived from different measurement procedures and/or different measurement principles into this common measurand is a major challenge for food allergen analysis. At present, measurement results are obtained using various types of immunoassays, PCR (polymerase chain reaction) or mass spectrometric methods. None of these measurement principles can directly measure (quantify, count or weigh) the ‘total protein derived from the allergenic food ingredient’. The entities actually measured are various epitopes, DNA fragments, individual proteins or peptides. Moreover, the agreed common measurand is a ‘sum parameter’, namely the sum of masses of various protein molecules from an ingredient, such as milk or egg, in the food sample. Therefore, the measurement data must be converted into the ‘mass of total protein of the allergenic ingredient per mass of food’, which requires a relationship between the parameters measured by the analytical method and the agreed measurand necessary for decision-making.

This manuscript outlines how food allergen contents derived from different analytical methods can be made comparable and expressed as the ‘mass fraction of total protein of the allergenic ingredient in food, in mg kg^−1^′ to enable consistent quantitative risk assessment. The case of ‘milk in cookies’ is illustrated as an example, while issues related to other allergens are discussed further.

## 2 The concept

Establishing meaningful decision thresholds or ranges for the allergen content of foodstuff to assess related consumer risks require comparable analytical data. In this context, comparability means that measurement results are metrologically traceable to the same reference, a common end-point against which values can be compared. When all measurement results are expressed as ‘mass of total protein of the allergenic food ingredient per mass of food’, the reported results become traceable to this reference.

Metrological traceability has been defined as “*the property of a measurement result whereby the result can be related to a reference through a documented unbroken chain of calibrations, each contributing to the measurement uncertainty*” (JCGM 200, 2012). However, the implementation of such a ‘chain of calibrations’ in allergen analysis is challenging due to the use of various surrogate analytes instead of proteins and the application of different analytical techniques in practice.

A specific challenge arises due to the definition of the common measurand. Similar to other sum parameters in food chemistry, such as ‘fat content’, one could harmonise the ‘what is measured’ in allergen analysis by agreeing on the ‘how to measure’. In metrology, such a protocol is called a ‘reference measurement procedure’ (JCGM 200, 2012). However, a metrological description of the method specifications for quantifying a food allergen could be challenging to implement by many field laboratories in the food control sector. Therefore, additional supporting tools are needed: reference materials for calibration, well-documented procedures, and conversion factors to transform data from the measured analytical target to the commonly agreed measurand.

In the early 90s, the community of clinical chemistry and laboratory medicine introduced the concept of ‘reference measurement systems’ (RMS) for analytes such as enzymes and other proteins. More recently, De Bièvre et al. (2011) have summarised various generic approaches for establishing metrological traceability of measurement results in chemistry and included several examples of reference measurement procedures. According to ISO 17511 (2020), an RMS consists of (i) a definition of the measurand, (ii) a reference measurement procedure, (iii) a reference material and (iv) one or more laboratories providing reference measurement services, so-called reference measurement laboratories.

**Fig. 1:**
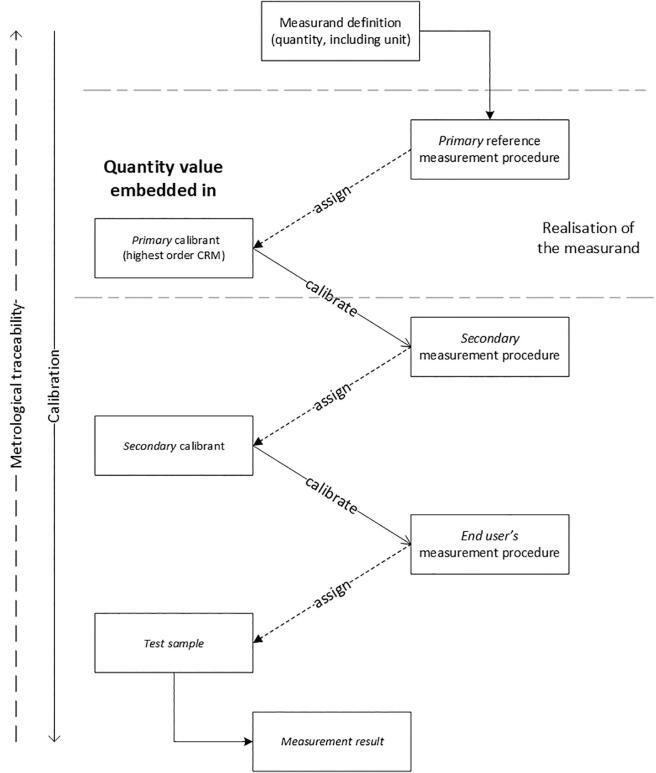
Suggested scheme for a calibration hierarchy and establishing metrological traceability.

The RMS for food allergen analysis proposed in this manuscript will allow the expression of results derived from different measurement procedures in a common measurand, such as ‘mass fraction of total protein of the allergenic ingredient in food’. This requires several conventions to be established and agreed upon by the scientific community. At first, a calibration hierarchy has to be developed to define the route (or metrological traceability chain) to be followed from the analytical data obtained by the measurement procedure used to the highest available metrological reference or end-point included in the proposed RMS and represented by the measurand in Fig. 1.

The practical realisation of the measurand, defined as ‘mass fraction of total protein of the allergenic ingredient in food’, is achieved by using a primary ‘reference measurement procedure’ for assigning a value for the measurand to an adequately homogeneous and stable material. This material may result in a certified reference material (CRM) with a well-established assigned property value with stated uncertainty (ISO Guide 30, 2015). This CRM would become the ‘primary calibrant’ with the highest metrological order containing the measurand of interest.

The implementation and execution of a reference measurement procedure are often time-consuming, expensive and difficult to perform because the objective is to obtain highly accurate results with the smallest achievable measurement uncertainties. That is why reference measurement procedures are usually developed and applied by only a few reference measurement laboratories. Such procedures are not designed for routine laboratories but serve as a standard against which other analytical procedures can be evaluated.

Unfortunately, reference materials (or materials labelled as such) have been extensively misused in the allergen community so far. For example, materials that were only characterised (and even certified) for the content of components not related to allergens were used as common controls for protein measurements. Similarly, laboratories used other matrix materials spiked with unrealistically large amounts of proteins or subjected to severe heat treatments (Abbott et al., 2010, Lacorn and Immer, 2011). Such practices were often rooted in misunderstandings regarding the proper measurand and/or the interrelation between the intended use of the reference material in the measurement process (e.g., calibration, trueness control, intra- or inter-laboratory quality controls) and the corresponding material characteristics.

The highest order CRM would serve as a calibrant for other ‘secondary measurement procedures’ also used outside the reference measurement laboratory and will allow the characterisation of the next order calibrants (see Fig. 1). It is important to remember that field laboratories may use a variety of procedures based on similar or different measurement principles. The ‘secondary calibrants’ used by the manufacturers of the allergen test kits or by the analytical laboratories for calibration and verification of the performance characteristics of their methods should have the characteristics of a reference material mentioned above.

In the concept illustrated by Fig. 1 the metrological traceability of the measurement result is established via a sequence of calibration and value assignment steps to reach the common reference, namely ‘the measurand’. As expected, each of these steps contributes to (and therefore increases) the measurement uncertainty of the final test result.

## 3 Case study: Milk

The metrological traceability and calibration hierarchy concepts for food allergen measurements presented above are applied hereafter to determine the ‘mass fraction of total milk protein in cookies’ (the measurand).

**Fig. 2:**
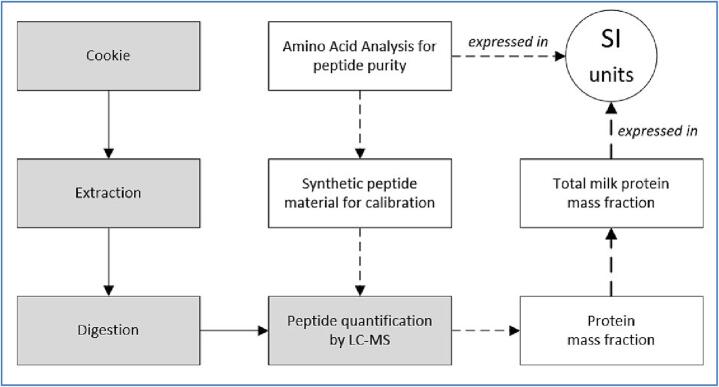
Workflow of the reference measurement procedure to determine the ‘mass fraction of total milk protein in cookies’, adapted from Martinez-Esteso et al. (2020).

The Joint Research Centre (JRC) of the European Commission developed a dedicated reference measurement procedure (Martinez-Esteso et al., 2020) for this measurand, based on the quantification of eleven peptides by liquid chromatography-mass spectrometry (see Fig. 2). In this context, Nitride et al. (2019) have optimised the extraction and digestion steps that were considered critical. In addition, Martinez-Esteso et al. (2020) demonstrated that complete protein digestion was achieved and that the variability of the extraction yield was within the uncertainty of the measurement results. While the metrological traceability of the reported result to the SI unit ‘mole’ was ensured by the well-characterised synthetic peptides used for calibration, certain conversion factors (conventions) were necessary to convert the peptide contents measured into the agreed measurand, namely ‘mass fraction of total milk protein in cookies’. Hence, the contribution of the protein molar masses and the protein compositions of milk described in the literature were considered when calculating the total (combined) uncertainty of the measurement results (Breidbach et al., 2022).

**Fig. 3:**
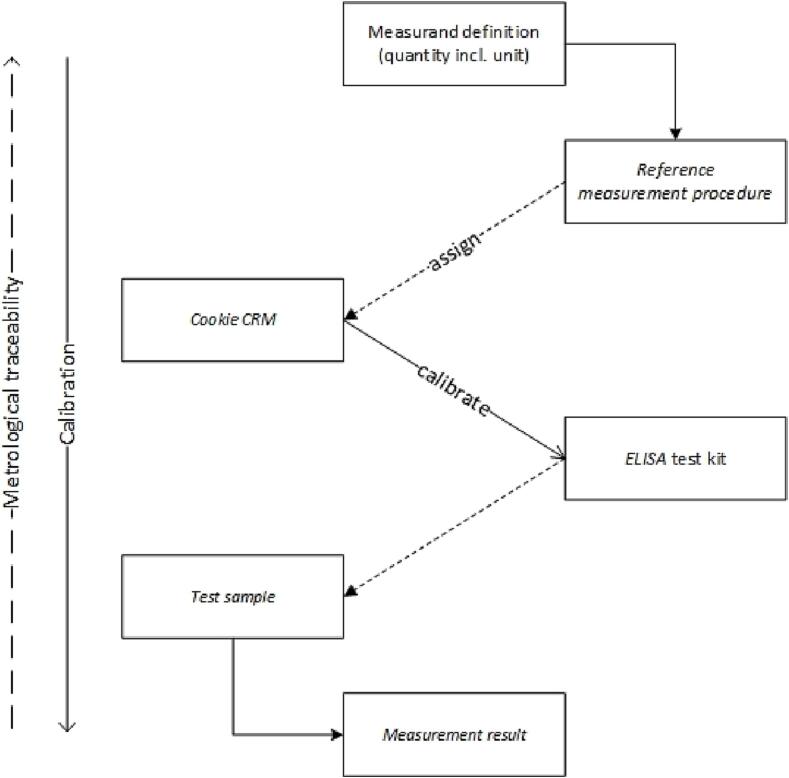
Metrological traceability chain for routine measurement results on the content of total milk protein in cookies as proposed here; reference measurement procedure as published by Martinez-Esteso et al. (2020).

Subsequently, this reference measurement procedure was successfully applied to assign the reference value for the allergen content in a proficiency test item (Cordeiro et al., 2021). It will be further used for the characterisation of a processed reference material to be certified for the ‘mass fraction of total milk protein in cookies’. The resulting CRM is intended to serve as the highest order CRM (see Fig. 1) for the analysis of matrix samples containing whole milk powder and should allow the calibration of other measurement procedures, such as an ELISA test kit (see Fig. 3). This should enable the reporting of measurement results for the content of milk protein in food from different routine tests expressed as the same measurand. Thereby, a meaningful comparison of measurement results would be possible.

The calibration hierarchy illustrated in Fig. 3 can be extended to other measurement tasks that involve a reference measurement procedure and its corresponding CRM.

## 4 Considerations for other allergens

The general concept of a calibration hierarchy depicted in Fig. 1 also applies to other food allergens and is necessary to ensure comparable measurement results. However, a better knowledge of the protein composition of various food ingredients and their potential biochemical modifications (altering their composition or structure) is required to derive reliable conversion factors that will also impact the uncertainty associated with the certified value of the primary CRM.

In this context, the case of total hen’s egg protein illustrates well the complexity of food allergen quantification. According to Kovacs-Nolan et al. (2005), the total mass of egg comprises approximately 12 % m/m of proteins, which are distributed in the egg yolk and the egg white, with a smaller portion in the shell and the membrane. Ovalbumin, ovotransferrin and ovomucoid are the three major components (ca. 77 % m/m) of the total protein in egg white, with ten other less abundant proteins mentioned in the literature. Similarly, the three main components (about 77 % m/m) of the total protein in egg yolk are spovitellenin (I-VI), ɑ–lipovitellin and β-lipovitellin. However, due to the limitations of the currently available analytical techniques, the accurate quantification of low-abundance proteins is not achievable.

Therefore, the sum approach outlined by Martinez-Esteso et al. (2020) for the milk ‘reference measurement procedure’ cannot be applied. However, an alternative approach using the conversion factors could be developed. For this, the allergen community needs to select and agree on several analytical markers for egg (e.g., ovalbumin and spovitellenin) and has to determine the corresponding conversion factors that should be used to quantify the mass fractions of ‘total egg protein’. The combined uncertainty associated with this approach would include contributions from the uncertainties of the conversion factors.

A challenge in selecting appropriate egg markers arises from the food industry’s standard practice of incorporating only egg white, instead of the whole egg, in their recipes. This could lead to an overestimation of the total egg protein content by a laboratory analysing such food samples with an egg-white specific ELISA test kit, as it may wrongly assume the presence of egg yolk (Nguyen et al., 2019). However, if the same laboratory would use an ELISA test kit targeting an egg yolk specific protein, they may conclude that no egg is present in the same food samples. Therefore, the food analyst must know the food ingredients (or potential contaminants) used.

When selecting the analytical target(s) for the food ingredient of concern or its relevant constituents, such as egg white and yolk, their stability under anticipated food processing conditions and their pertinent analytical behaviour must also be considered. For instance, research has demonstrated that egg proteins extracted from tempered chocolate exhibit a lower affinity to antibodies generated with raw egg proteins when compared to antibodies produced against boiled egg white, boiled ovomucoid and boiled ovalbumin (Khuda et al., 2015). This indicates that thermal processing may affect the design and application of immunological methods for protein quantification.

To sum up, additional studies are necessary to establish a correlation between a measurable quantity and the common measurand based on the parameter ‘total protein’ for the other food allergens. The scientific community needs to investigate further the protein composition of the relevant food ingredients, considering their biological variability, and identify relevant analytical targets that could withstand different food processing conditions. This data would enable reference measurement laboratories to develop the necessary reference measurement procedures and to produce the corresponding CRMs essential for establishing customised RMSs.

## 5 Conclusions

It is essential to establish common end-points for the metrological traceability chains for each allergen measurement to address the current issue of non-comparable data on the allergen content of food. These end-points can be realised via dedicated reference measurement procedures (executed by reference measurement laboratories) and tailored CRMs. The design and application of such a RMS has been successfully demonstrated for the potentially allergenic food ingredient ‘milk’ using a dedicated multiple protein calibration regime with an LC-MS approach.

Implementation of a common allergen measurand defined as ‘mass fraction of total protein of the allergenic ingredient in food’ is challenging. It requires in-depth knowledge of the structures of the targeted proteins, their biological variability (e.g. seasonal, geographical or animal species), and their quantitative composition in the food ingredients. Hence, systematic research should be conducted before developing similar reference measurement systems for other food allergens.

A concerted effort is necessary between reference and testing laboratories, reference material producers and manufacturers of commercial allergen test kits to ensure that the approach described in this manuscript is effectively implemented and that the measurement results for food allergen analysis are comparable.

## CRediT authorship contribution statement

**Elena Cubero-Leon:** Conceptualization, Writing – original draft. **Hendrik Emons:** Supervision, Conceptualization, Writing – review & editing. **Gavin O'Connor:** Conceptualization, Writing – review & editing. **Jørgen Nørgaard:** Writing – review & editing. **Piotr Robouch:** Conceptualization, Writing – review & editing.

## Declaration of Competing Interest

The authors declare that they have no known competing financial interests or personal relationships that could have appeared to influence the work reported in this paper.

## Data Availability

No data was used for the research described in the article.

## References

[b0005] Abbott M., Hayward S., Ross W., Godefroy S.B., Ulberth F., Van Hengel A.J., Delahaut P. (2010). Validation Procedures for Quantitative Food Allergen ELISA Methods. Community Guidance and Best Practices. Journal of AOAC International.

[b0010] Ballmer-Weber B.K., Fernandez-Rivas M., Beyer K., Defernez M., Sperrin M., Mackie A.R., Mills E.N.C. (2015). How much is too much? Threshold dose distributions for 5 food allergens. Journal of Allergy and Clinical Immunology.

[b0015] Breidbach A., Nørgaard J.V., Cubero-Leon E., Martinez Esteso M.J.. (2022). Assignment of a Reference Value of Total Cow’s Milk Protein Content in Baked Cookies Used in an Interlaboratory Comparison. Foods.

[b0020] Cordeiro F., Cubero‑Leon E., Nørgaard J., Martinez‑Esteso M.-J., Brohée M., Breidbach A., Emons H. (2021). Total cow’s milk protein in cookies: the first interlaboratory comparison with a well‑defined measurand fit for food allergen risk assessment. Accreditation and Quality Assurance.

[b0025] De Bièvre P., Dybkær R., Fajgelj A., Hibbert D.B. (2011). IUPAC Technical Report Metrological - traceability of measurement results in chemistry: Concepts and implementation. Pure and Applied Chemistry.

[b0030] ISO Guide 30:2015. Reference materials — Selected terms and definitions. International Organization for Standardization, Geneva, Switzerland.

[b0035] ISO 17511:2020. In vitro diagnostic medical devices — Requirements for establishing metrological traceability of values assigned to calibrators, trueness control materials and human samples. International Organization for Standardization, Geneva, Switzerland.

[b0040] JCGM 200:2012. Joint Committee for Guides in Metrology. International Vocabulary of Metrology – Basic and General Concepts and Associated Terms (VIM, 3rd edition), BIPM, Sevres. https://bit.ly/3uSWZ9H, accessed on 04/04/2023.

[b0045] Khuda S.E., Jackson L.S., Fu T.-J., Williams K.M. (2015). Effects of processing on the recovery of food allergens from a model dark chocolate matrix. Food Chemistry.

[b0050] Kovacs-Nolan J., Phillips M., Mine Y. (2005). Advances in the value of eggs and egg components for human health. Journal of Agricultural and Food Chemistry.

[b0055] Lacorn M., Immer U. (2011). Allergen determination in food: Reference materials and traceability of results. Accreditation and Quality Assurance.

[b0060] Martinez-Esteso M.J., O’Connor G., Nørgaard J., Breidbach A., Brohée M., Cubero-Leon E., Emons H. (2020). A reference method for determining the total allergenic protein content in a processed food: The case of milk in cookies as proof of concept. Analytical and Bioanalytical Chemistry.

[b0065] Nguyen A.V., Williams K.M., Ferguson M., Lee D., Sharma G.M., Do A.B., Khuda S.E. (2019). Enhanced quantitation of egg allergen in foods using incurred standards and antibodies against processed egg in a model ELISA. Analytical Chimica Acta.

[b0070] Nitride C., Nørgaard J., Omar J., Emons H., Martinez Esteso M.J., O’Connor G. (2019). An assessment of the impact of extraction and digestion protocols on multiplexed targeted protein quantification by mass spectrometry for egg and milk allergens. Analytical and Bioanalytical Chemistry.

[b0075] O'Connor, G. & Ulberth, F. (2017) Joint DG SANTE and DG JRC Workshop - Harmonisation of approaches for informing EU allergen labelling legislation, JRC108259, available from https://bit.ly/2xnNl4i, accessed on 04/04/2023.

[b0080] Regulation (EC) No 178/2002 of the European Parliament and of the Council of 28 January 2002 laying down the general principles and requirements of food law, establishing the European Food Safety Authority and laying down procedures in matters of food safety. *Official Journal*, L 031.

[b0085] Regulation (EU) No 1169/2011 of the European Parliament and of the Council of 25 October 2011 on the provision of food information to consumers. *Official Journal*, L 304.

[b0090] Remington B.C., Baumert J., Blom W.M., Bucchini L., Buck N., Crevel R., Walker M. (2022). Allergen quantitative risk assessment within food operations: Concepts towards development of practical guidance based on an ILSI Europe workshop. Food Control.

[b0095] Soon J.M., Manning L. (2017). “May Contain” Allergen Statements: Facilitating or Frustrating Consumers?. J Consumer Policy.

[b0100] WHO/FAO (2021a). Ad hoc Joint FAO/WHO Expert Consultation on Risk Assessment of Food Allergens Part 2: Review and establish threshold levels in foods of the priority allergens. Issued on August 20, 2021. Available from https://bit.ly/3hnflvW, accessed on 04/04/2023.

[b0105] WHO/FAO (2021b). Ad hoc Joint FAO/WHO Expert Consultation on Risk Assessment of Food Allergens Part 3: Review and establish precautionary labelling in foods of the priority allergens. Issued on December 13, 2021. Available from https://bit.ly/3zCvOC9, accessed on 04/04/2023.

